# How Does Social Comparison Influence Chinese Adolescents’ Flourishing through Short Videos?

**DOI:** 10.3390/ijerph19138093

**Published:** 2022-07-01

**Authors:** Sijia Guo, Kun Bi, Liwei Zhang, He Jiang

**Affiliations:** 1 College of Public Administration and Humanities, Dalian Maritime University, Dalian 116026, China; sijiaguo@dlmu.edu.cn; 2 School of New Media, Peking University, Beijing 100871, China; bikun@pku.edu.cn; 3 School of Public Administration, Jilin University, Changchun 130012, China; 4 Department of Social Psychology, Nankai University, Tianjin 300350, China; jhpsy@mail.nankai.edu.cn

**Keywords:** adolescents, flourishing, social comparison, social media use

## Abstract

Flourishing indicates one’s emotional status and functioning level and is essential for adolescents’ further development. Adolescents’ social media use has been rising, with various potential effects on their development. Therefore, in this study, we shifted the emphasis from a traditional deficit-based approach to a strength-based approach by exploring how social comparison and social media usage influence Chinese adolescents’ flourishing. Altogether, 786 Chinese adolescents aged 12–19 years completed a self-report questionnaire. The results indicate that (1) both social media social comparison of ability (SCA) and social media social comparison of opinion (SCO) have no significant effect on Chinese adolescents’ flourishing; (2) integration into social routine has a positive indirect effect on the relationship between social media social comparison and flourishing; and (3) social integration and emotional connection negatively affect the relationship between social media social comparison and flourishing. These findings highlight the interaction between social media social comparison and social media usage. Furthermore, the results of this study clarify that the potentially harmful effect of social media usage on adolescents’ flourishing is not determined by the frequency or time spent on using social media, but how much adolescents are connected to or invested in social media.

## 1. Introduction

Adolescents in a transitional period deal with numerous challenges, such as exploring and committing to identities, resulting in disparate effects on their mental health. Therefore, an urgent public health issue is to understand the protective factors for positive social adaptation during this process. In recent years, social media use has become an ever-increasing phenomenon embedded within adolescents’ lives. YouTube, Instagram, and Snapchat are the most popular online platforms among U.S. teens. Specifically, approximately 85% of U.S. teens use YouTube, 72% use Instagram, and 69% use Snapchat [1].

Similarly, according to the latest report from the China Internet Network Information Center, 183 million Chinese minors use the Internet, and the Internet availability rate among Chinese minors reaches 94.9% [2]. Short videos rank second among all social media platforms in usage rate [2]. In other words, social media platforms, especially short videos, have exerted effects on Chinese adolescents.

Numerous studies have focused on the relationship between social media usage and adolescents’ development. Some studies pointed out that social media use yielded a series of risks for young people’s development, such as cyber-bullying, Internet addiction, identity confusion, and social isolation [3,4,5]. Nevertheless, other studies have highlighted the positive benefits of social media: it provides a channel for adolescents to engage in self-disclosure, access multi-dimensional online resources, or even increase their social capital [6,7]. Although there are numerous studies focusing on the effects of social media, the results are still ambiguous. Compared with the traditional deficit-based approach, in this study, we concentrate on the strength-based approach by exploring how social media influences Chinese adolescents’ flourishing.

Flourishing is a new perspective on well-being that stresses not only focusing on the weakness of adolescents, but also highlighting the positive aspects of adolescents. Flourishing integrates multiple facets combining hedonism, psychological functioning, and social functioning [8]. Hedonism represents one’s degree of happiness and life satisfaction and is the basis of well-being. Psychological functioning refers to one’s intrinsic capability to actualize self-growth, which comprises six comprehensive dimensions: self-acceptance, positive relations with others, autonomy, environmental mastery, purpose in life, and personal growth [9]. Additionally, social functioning indicates social well-being, including social integration, social acceptance, social contribution, social actualization, and social coherence [8]. Therefore, flourishing is an integrative and solid viewpoint related to personal affection and functioning, a necessary antecedent determinant for adolescents’ development. As such, burgeoning studies have gradually shed light on the effects of flourishing. For example, Nelson and Padilla-Walker [10] found that flourishing adolescents showed better performance in social adaption and personal functioning when entering emerging adulthood. Herein, flourishing is a new perspective on adolescent studies compared with the deficit orientation, and it comprehensively examines the individual’s well-being by integrating emotion and functioning. From this perspective, in this study, we examine the flourishing of Chinese adolescents.

### 1.1. Social Media Social Comparison and Flourishing

Social comparison refers to the process of comparing oneself with others in two forms: social comparison of ability (SCA) and social comparison of opinion (SCO) [11]. Specifically, SCA entails performance and achievement comparisons that are preferable to being judgmental and competitive. Conversely, SCO is related to comparing values, beliefs, and thoughts and involves communicating with others, learning from social norms, and modifying one’s value system [12,13]. Although both SCA and SCO are the sub-dimensions of social comparison orientation, SCA differs from SCO. The former underlines judgement or comparison, while the latter typically concerns the similarities and differences in opinions among people or groups. In view of the definition and nature of social comparison, extant studies link social comparison orientation with social media, as social media creates a venue and provides opportunities for individuals to engage in online activities, such as interacting and comparing themselves with others [14].

As social media use becomes ubiquitous, researchers have gradually focused on how online social comparison influences one’s well-being and development. For instance, Lee [15] found a negative relationship between social comparison on Facebook and personal affection, primarily when individuals engage in upward comparisons. Additionally, Vogel et al. [16] proposed that social media users were inclined to proceed upward with a social comparison with others on social networking sites, which negatively influenced their self-perception and psychological functioning, including experiencing low self-esteem and a sense of competition. Thus, it can be seen that some studies argue that social comparison on social media have adverse effects on personal well-being [17,18,19]. Nevertheless, other scholars have elaborated that upward social comparison, as a motivator, helps the individual set a goal and endeavor to actualize self-improvement [12]. Additionally, downward social comparison also exists on social media, making individuals feel more satisfied with life [20]. However, the empirical studies on the association between social comparison and personal well-being do not differentiate the sub-dimensions of ability- or opinion-based social comparison. Most of the studies, in fact, focus on the effects of social comparison of ability, resulting in the observations of only upward or downward social comparison [21]. Furthermore, several studies determined that instead of contributing to negative emotions, social comparison of opinion could promote life satisfaction [21]. Therefore, in this study, we intended to explore how online social comparison influences Chinese adolescents’ flourishing by separating it into two dimensions.

### 1.2. Social Media Use Integration as a Mediator

Social media allows users to interact with people who might be acquaintances or strangers offline and access diverse information, which leads to the prolonged time spent online. Most prior studies refer to social media use integration in terms of frequency or intensity. However, Ellison [6] postulated that the nature of social media not only indicates the usage frequency, but also involves a user’s emotional connection to social media and the integration of its use in daily life. Thus, social media use integration should include two dimensions, which are users’ behaviors and routines and the emotional connection to social media usage.

Some studies postulated that online social comparison influences social media use integration, as social comparison orientation is an inherent personal characteristic that influences an individual’s behaviors in daily life. Specifically, social media platforms create more opportunities for individuals with high social comparison orientation to compare themselves with others, which increases the intensity of one’s social media usage [15]. Offline, individuals prefer to compare themselves with people around them, such as friends or colleagues, while social media on the Internet allows people to compare upward or downward. From this standpoint, people with high social comparison orientations are inclined to engage in social media usage. Vogel et al. [16] supported this idea by conducting research with 145 undergraduates from the U.S. Other studies point out that the relationship between social media social comparison and social media use integration is bidirectional. In other words, if individuals frequently engage in social media use, they are more likely to be exposed to social comparisons with other people and information [15]. According to Festinger’s social comparison theory [11], comparison with others is a spontaneous motivation among individuals. From this perspective, in this study, we regard online social comparison as a personal characteristic, which means that social media social comparison is an antecedent of social media use integration.

Meanwhile, most studies focus on the influence of social media use on one’s health-related aspects, such as affection status, psychological functioning, and social well-being. One argument states that spending time on social media is detrimental to one’s long-term well-being. For example, Brooks [22] applied the distraction–conflict theory to examine the relationships between social media usage and self-efficacy as well as social media usage and personal well-being. Additionally, they found that social media usage is negatively related to performance and happiness. Contrarily, the other argument from empirical studies considers that social media usage in daily life is positively associated with a positive mood for individuals with a relatively small social network [23,24]. Consequently, results on social media usage and well-being remain inconclusive. Although the association between social media usage and personal well-being remains, the mechanism linking social media social comparison, social media usage, and flourishing among rural Chinese adolescents needs to be explored.

### 1.3. The Present Study

Flourishing is a vital determinant of adolescents’ development and has been thoroughly investigated in multiple disciplines, including social work, family studies, and positive psychology. One’s flourishing is intertwined with various layers, including personal characteristics, social contexts, and even cultural contexts. Social media, as a ubiquitous tool, significantly influences people. From this perspective, in this study, we aim to explore how social media influences Chinese adolescents’ flourishing. There are numerous social media platforms for adolescents to engage in, yielding different impacts. Specifically, we explore the effects of short videos on social media (e.g., TikTok, Kuai Shou, etc.), in line with the usage ranking from the newest national report from China [2].

Furthermore, social comparison theory provides a theoretical lens for understanding the effects of social media on adolescents’ flourishing. As mentioned above, comparison with others is an inherent characteristic of human beings, and social media offers more opportunities for individuals to consciously or unconsciously compare themselves with others. Therefore, how social media social comparison orientation influences individuals’ flourishing is worth exploration, especially for adolescents in a transitional period who explore and commit to identities by interacting with others. Additionally, the empirical studies also postulated that social comparison orientation influences one’s motivation to engage in social media. Accordingly, in this study, we aim to explore the relationship between social media social comparison and adolescents’ flourishing, as well as the mediated effects of social media use integration. Furthermore, as social comparison includes ability-based and opinion-based comparison, and social media use integration includes routine use in social media and emotional investment in social media, in this study, we examine the effects of each dimension specifically.

**Figure 1:**
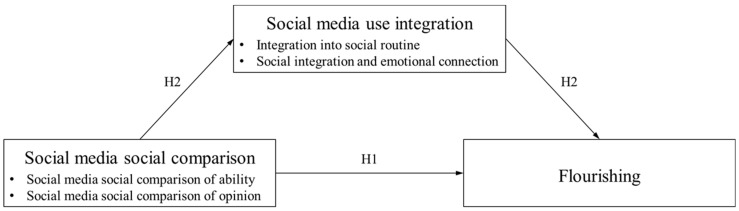
The theoretical model and research hypotheses.

Accordingly, we propose the following hypotheses (see Figure 1):

**Hypothesis** **1**. *Social media social comparison (i.e., ability and opinion) negatively influences Chinese adolescents’ flourishing.*

**Hypothesis** **2**. *Social media use integration (i.e., integration into social routine and social integration and emotional connection) negatively mediates the relationship between social media social comparison (i.e., ability and opinion) and flourishing among Chinese adolescents.*

## 2. Method

### 2.1. Participants

A total of 800 participants were recruited from two junior and two senior high schools in Liaoning and Sichuan, China. After removing 14 invalid samples, 786 questionnaires were obtained, with a response rate of 98%. Among the participants, 51% were boys and 49% were girls, ranging from 12 to 19 years of age (see Table 1). Moreover, approximately 79% of the participants’ parents were in the state of being married (i.e., not divorced, separated, or widowed). Most participants’ academic grade rankings were lower than 20% in their class. Furthermore, there were notable differences between the hours spent watching short videos between weekdays and weekends. More specifically, nearly half of the participants did not watch short videos during weekdays, while the majority of the participants spent 0.5 to 3 h watching short videos on weekends. Almost 93% of the participants slept for 5 to 8 h per day (see Table 1). The data were collected at the end of 2021. Permissions were obtained from the schools. Furthermore, written informed consent was obtained from all participants and their parents or guardians. The study’s voluntary nature, anonymity, and confidentiality were ensured. The participants completed a self-report questionnaire with support from school teachers. The Institutional Review Board of College of Public Administration and Humanities at Dalian Maritime University approved this study.

### 2.2. Measurement

Social media social comparison. Social media social comparison was measured using the Social Media Social Comparison (SMSC) scale [25]. This scale originates from the Iowa-Netherlands Comparison Orientation Measure (INCOM), widely applied to measure social comparison activities on social media [12]. This study has two subscales: Social Media Social Comparison of Ability (SMSC-Ability) and Social Media Social Comparison of Opinion (SMSC-Opinion). Sample questions include “On social media, I compare what I have done with others as a way to find out how well I have done something” and “On social media, I try to know what others in a similar situation would do” for the two sub-scales, respectively. Moreover, participants were required to rate how they compared themselves with others using social media on a 5-point Likert scale. A high score indicates a high level of social comparison when using social media. In the present study, both the SMSC-Ability and SMSC-Opinion scales had good reliability, with Cronbach’s alpha values of 0.69 and 0.83.

Social media use integration. Ten items of the social media use integration scale (SMUIS, [26]) were adapted to measure social media use integration. The original items concentrated on use integration on Facebook, but we intended to measure use integration on social media. Therefore, we modified the original items by replacing “Facebook” with “social media platforms”. An example item is “I get upset when I cannot log on to social media.” This scale comprises two subscales: Integration Into Social Routine (ISI) and Social Integration and Emotional Connection (SIEC). Items were rated on a 5-point Likert scale; simultaneously, a higher score indicated a high level of use integration. Cronbach’s alpha values for the two sub-scales were 0.67 and 0.82.

Flourishing. We applied the 14-item Mental Health Continuum-Short Form Scale to measure flourishing (MHC-SF, [8,27]). An example item is “How often did you feel that you belonged to a community”. The items were rated on a 5-point Likert scale. A higher score indicated a higher level of flourishing. The measure showed good reliability in this study, with Cronbach’s alpha of 0.84.

Self-esteem. The six-item State Self-Esteem Scale (SSES-6) was used to measure self-esteem [28]. An example item is “I am worried about what other people think of me”. A 5-point Likert scale was used to rate the items, and a higher score implied a lower level of self-esteem. The measure showed good reliability in this study, with Cronbach’s alpha of 0.76.

Demographic details. Participants answered questions about their demographic backgrounds, including sex (i.e., 0 = girls, 1 = boys), age, parental marital status (i.e., 0 = unmarried, 1 = married), hours spent watching short videos on weekdays and weekends, academic grade ranking (i.e., 0 = below the top 20%, 1 = within the top 20%), and sleeping hours.

### 2.3. Statistical Analysis

We applied Stata 17.0 for zero-order correlations and Structural Equation Modeling (SEM) with maximum likelihood estimation. SEM is suitable for a multiple-mediator model [29]. Furthermore, the Monte Carlo method was used to measure the indirect effects of mediation, as this method is appropriate for SEM [30].

According to the theoretical model (Figure 1), a path model postulates that social media social comparison (i.e., SMSC-Ability and SMSC-Opinion) predicts social media using integration (i.e., integration of social routines and social integration and emotional integration) and flourishing. Social media use integration (i.e., integration of social routines, and social integration and emotional integration) mediates social media social comparison and flourishing. We executed the correlation analysis first, followed by a two-step SEM approach to examine the relationships among the above variables. The first step examined the acceptability of the fit of the measurement model, and the second step tested the structural models. With the present study, we aim to understand general flourishing rather than the various states of its dimensions. Thus, flourishing was modeled as a latent variable.

Additionally, bootstrapping with 5000 replications was performed to test the significance of the direct and indirect relationships among the variables. Particularly, this method was employed to improve the representative power of the sample. When the results showed 95% confidence intervals (CIs) with non-zero values, the standardized estimates of the relationships were statistically significant [31,32].

## 3. Results

### Measurement Model

**Figure 2:**
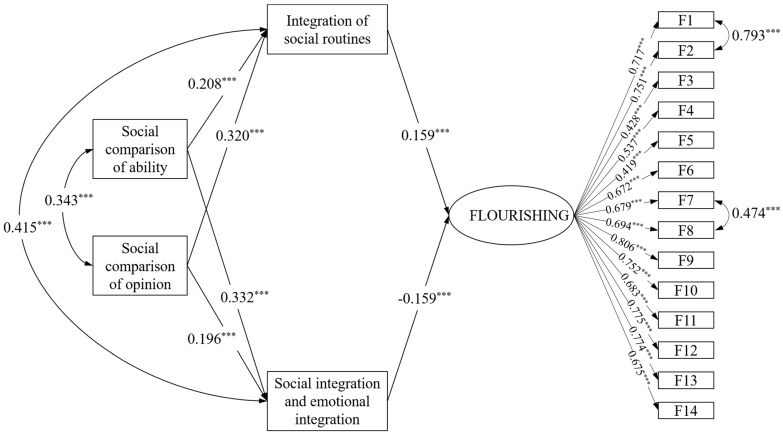
The standardized SEM results of social media social comparison, social media use integration, and flourishing. Note: Non-significant links, control variables, and error terms are omitted from the figure. *** *p <* 0.01.

Figure 2 and Table 2 show the SEM results. In line with Hu and Bentler [33], the model yielded a good model fit: $\chi^{2}/df=2.10<3$, comparative fit index (CFI) = 0.92 > 0.90, Tucker–Lewis index (TLI) = 0.90 > = 0.90, and root mean square error of approximation (RMSEA) = 057 < 0.06. H2 was partially supported in that SCA positively predicted both integration of social routines and social integration and emotional integration (β=0.208, p<0.00; β=0.332, p<0.00). H2 was also supported in that SCO showed significant effects on integration of social routines (β=0.320, p<0.00) and social integration and emotional integration (β=0.196, p<0.00). The results demonstrated that neither SCA nor SCO had significant effects on flourishing. The Monte Carlo method (5000 replications) showed partial support for H2 in that, although the effect size was small, the indirect effects of integration of social routines and social integration and emotional integration mediating between SCA and flourishing were 03 (95% [CI] = 0.005 to 0.007, <0.05) and −0.053 (95% [CI] = −0.102 to −0.011). Meanwhile, H2 was partially supported in that the indirect effects of integration of social routines and social integration and emotional integration mediating between SCO and flourishing were 05 (95% [CI] = 0.009 to 0.097, <0.05) and −0.03 (95% confidence interval [CI] = −0.065 to −0.006, <0.05).

## 4. Discussion

In this study, we employed social comparison theory to examine how social media social comparison influences Chinese adolescents’ flourishing through short videos. Several results generated from this study are worthy of discussion.

First, in terms of the first hypothesis, we found no significant relationship between SCA and flourishing among Chinese adolescents or between SCO and flourishing among Chinese adolescents. The debate on whether online social comparison promotes or impairs adolescents’ well-being and development has persisted for several years. The opposing view was prominent in research in the 2010s, whereas recent research stresses the positive impacts of social media social comparison, such as improvements in inspiration [34]. However, the results of this study are inconsistent with the prior dominant views. This discrepancy in the findings may be attributed to the following reasons. Primarily, most users are passive in engaging with short videos to meet their needs for entertainment, information, social interaction with others, etc. [35]. Likewise, some studies have shown that, unlike other social media platforms, such as WeChat, newsfeeds from short videos are not based on who one follows [36]. Accordingly, users who watch short videos tend to search for objects that are humorous or easy to imitate. Therefore, when people browse short videos in a short amount of time, they would not like to watch them carefully. In other words, people are not distracted and are affected by social comparison orientations. In the same vein, the results of other empirical studies are supportive. For instance, Yang [37] found that social comparison orientation did not affect personal well-being when only browsing rather than reading others’ information carefully.

Second, we found the mechanism by which social media use routines and emotional investment influenced the relationship between SCA and Chinese adolescents’ flourishing, and between SCO and Chinese adolescents’ flourishing. Specifically, integration into social routines shows a positive indirect effect, while social integration and emotional connection display a negative indirect effect, which partially supports the second hypothesis. Both SCA and SCO positively affected integration into social routines, and integration into social routines positively affected Chinese adolescents’ flourishing. It is reasonable to explain the relationship between social comparison (including SCA and SCO) and ISR, as social media is the place where adolescents could gain more information and access to more people. For adolescents, they are in a transitional stage in which they would like to compare themselves with their peers to exercise self-evaluation and constantly revise their self-perception [15,25]. Herein, adolescents with a high social comparison orientation are prone to engage in social media routines frequently in daily life, as they are more likely to be aware of the value of social media. Vogel et al. [38] also found a positive correlation between social comparison orientation and social network sites (SNSs) use intensity. Furthermore, due to the characteristics of social media, its functioning is to contact others who are homogeneous and heterogeneous, search for various forms of support, and share oneself with others. From this perspective, empirical studies have pointed out that one of the benefits of social media is to maintain personal social capital. Thus, using social media frequently in daily life contributes to expanding social networks and enhancing social trust and civic engagement, which, in turn, facilitates one’s well-being [6,39]. Therefore, integration into social media has a positive indirect effect on the association between social comparison on social media and adolescents’ flourishing in China.

We found that both SCA and SCO showed positive effects on social integration and emotional integration, while social integration and emotional integration were negatively associated with Chinese adolescents’ flourishing. Emotional connection to social media has been the focus of only a few studies, yet it is an essential dimension of social media usage [26]. This study’s findings shed light on the nature of social media usage, highlighting the individual’s routine behavior and emotional involvement when using social media. Adolescents with higher social comparison orientation levels prefer to emotionally compare their abilities and opinions with others to achieve self-evaluation and commit to self-identities. Therefore, a higher social comparison orientation facilitates the individuate to invest more emotion and feeling into social media usage.

Meanwhile, we found that emotional connections with social media were negatively related to Chinese adolescents’ flourishing. An emotional connection to social media indicates that the individual considers social media necessary in social relationships. People with high emotional connections to social media are more likely to report negative emotional symptoms and functioning. Regarding this finding, several previous studies have arrived at similar results. For instance, Bekalu et al. [40] conducted a study by recruiting 1027 American adults to examine how social media use influences social well-being and found that an emotional connection to social media use was negatively associated with all dimensions of social well-being. Meanwhile, Woods and Scott [41] also demonstrated that an emotional connection to social media was detrimental to one’s sleep and well-being in a sample of 467 adolescents. Herein, one needs to be aware that an emotional connection to social media could potentially generate harmful results for adolescents, even with norms of social media use behavior.

In addition, we also found that Chinese adolescents’ flourishing increased with high self-esteem and more sleeping hours, which is consistent with the findings of empirical studies [42,43]. Adolescents with high self-esteem generally have stable identities, adaptive behaviors, and high-quality relationships, facilitating their well-being. Sleeping is an essential antecedent to adolescents’ physical and emotional well-being. Most studies proposed that adolescents normally typically need from 6 to 8 h of sleep per night, and adequate sleep hours are positively related to adolescents’ attention, motivation, and adaptive behaviors [44]. Moreover, we found that adolescents whose parents live together experience high levels of flourishing, demonstrating the importance of family in adolescents’ development.

The implications of this study are twofold. On the one hand, regarding the theoretical framework, we focused on adolescents’ flourishing using a strength-based approach rather than the traditional deficit-based approach. Hence, this study expands and complements empirical research on social media and adolescents’ well-being. Additionally, we applied the social comparison theory to assess social media social comparison, social media use, and Chinese adolescents’ flourishing. Notably, we examined the dimension of social comparison and social media usage and found that integration into social routines had a positive indirect effect. Contrastingly, social integration and emotional integration showed adverse indirect effects. This study enriches the empirical research on social media usage and simultaneously extends our understanding of the relationship among social media social comparison, social media use, and Chinese adolescents’ flourishing.

On the other hand, the results of this study also have several practical contributions. Social media is widely used among adolescents, and this study sheds light on the potential effects of social media on adolescents’ flourishing. The findings could attract the attention of parents, school teachers, social workers, and even public health professionals to design or strengthen interventions guiding adolescents to use social media rationally and cultivate control and self-regulation skills [44].

Considering the special situation of China, this study has some implications to promote adolescents’ flourishing from the perspective of the government, as well. First, it is a vital responsibility of the government to ensure a harmonious environment for adolescents. In this sense, the government ought to offer an institutionalized regulation for managing social media content to deliver adolescents more positive information. Meanwhile, the family, community, and government should establish a collaborative network to guide adolescents on how to use social media correctly.

This study has several limitations that should be addressed. Primarily, this is a cross-sectional study, which means that causality associations among the variables could not be generated. Furthermore, we only collected data from Liaoning Province and Sichuan Province, which means that we should be cautious about concluding generalized results. Longitudinal, panel, or experimental research should be conducted in the future. Additionally, although this study concerns how social comparison and social media use influence Chinese adolescents’ flourishing, we did not consider cultural values, social media use styles (e.g., active and passive use), or the characteristics of adolescents. Future studies could include other variables closely related to adolescents’ development to establish a more comprehensive model.

## 5. Conclusions

In this study, we investigated how social media influences Chinese adolescents’ flourishing. Specifically, we explored the relationship among social comparison, social media use, and adolescents’ flourishing. We found that social media social comparison did not significantly affect Chinese adolescents’ flourishing until social media use integration exerted a mediated effect. Furthermore, we found that the two dimensions of social media use integration, namely integration into social routines and social and emotional integration, showed reverse mediated effects. This study offers several theoretical and practical contributions to the literature. First, it adopts a positive perspective to stress the underlying mechanism between social media and adolescents’ flourishing. Second, it emphasizes that emotional connections with social media are more important than daily routines with social media. Finally, the findings of this study also provide practical suggestions for parents and school teachers regarding guidance and education.

## Figures and Tables

**Table 1:** Percentages of demographic characteristics (*n* = 786).

Characteristics	Percentage (%)
**Gender**	
Female	49%
Male	51%
**Parental Marital Status**	
Not married	21%
Married	79%
**Academic Grade Ranking**	
Lower than the top 20%	73%
Top 20%	27%
**Hours watching short videos on weekdays**	
NA	46%
0.5–1.5 h	38%
2–3 h	10%
>3 h	6%
**Hours watching short videos on weekends**	
NA	23%
0.5–1.5 h	51%
2–3 h	18%
>3 h	8%

**Table 2:** Standardized SEM results.

Predictors			->SMSC-A			->SMSC-O		->ISR		->SIEC		->FLO		
SMSC-A								0.21 ***		0.33 ***		−0.07		
SMSC-O								0.32 ***		0.20 ***		0.08		
ISR												0.16 ***		
SIEC												−0.16 ***		
**Control Variables** Self-esteem			0.26 ***			0.15 ***		0.00		0.08		−0.33 ***		
Male			0.07			0.01		−0.00		0.08		0.04		
Married			0.07			−0.04		−0.01		−0.02		0.11 *		
Age (years)			−0.00			0.04		0.03		−0.02		−0.05		
Academic ranking—upper 20%			0.06			−0.00		0.03		0.02		0.10		
SVWD			0.00			−0.07		0.01		−0.00		−0.07		
SVWE			−0.00			0.05		0.24 ***		0.25 ***		0.05		
Sleeping hours			−0.10			−0.09		−0.08		0.00		0.16 ***		
Factor loadings for indicators of the latent variable														
Flourishing->	F1	F2	F3	F4	F5	F6	F7	F8	F9	F10	F11	F12	F13	F14
Factor loading	0.72 ***	0.75 ***	0.43 ***	0.54 ***	0.42 ***	0.67 ***	0.68 ***	0.69 ***	0.81 ***	0.75 ***	0.68 ***	0.78 ***	0.78 ***	0.67 ***
Model-fit index														
$\chi^{2}/df=2.10$														
RMSEA = 0.057, with 90% CI = (0.05−0.06)														
CFI = 92; TLI = 90														

Note *** p < 0.01, * p < 0.5.

## Data Availability

The data used in this research are available on request from the corresponding author. The data are not publicly available due to restrictions.
